# Microfracture of chondral lesions of the glenohumeral joint

**DOI:** 10.4103/0973-6042.44142

**Published:** 2008

**Authors:** Martyn Snow, Lennard Funk

**Affiliations:** Department of Orthopaedics Wrightington Hospital, Lancashire, England, UK

**Keywords:** Chondral lesion, glenoid, humeral, microfracture

## Abstract

**Objective:**

To determine if microfracture is successful in treating chondral lesions of the shoulder.

**Design:**

Case series.

**Setting:**

Tertiary referral practice.

**Patients:**

From June 2005 to November 2006, eight patients underwent shoulder arthroscopy with arthroscopic microfracture to treat full-thickness chondral lesions of less than 4 cm^2^ size. The study group consisted of six men and two women. The mean age at surgery was 37 years (range: 27–55 years).

One patient (12.5%) had an isolated chondral defect and seven patients (87.5%) had associated conditions treated simultaneously: two patients had arthroscopic subacromial decompressions, two had capsular plications for multidirectional instability, and three had anterior stabilization done (one with an associated superior labrum anterior to posterior repair and one with repair of a small rotator cuff tear). Five patients had humeral head defects and three had glenoid defects.

**Intervention:**

Microfracture.

**Main outcome measures:**

Constant score and Oxford score.

**Results:**

The mean follow-up period was 15.4 months, with a range of 12–27 months. The mean preoperative Constant score was 43.88 (range: 28–70) and at final follow-up the mean Constant score was 90.25 (range: 85–100); this difference was significant (*P*<0.005). The mean preoperative Oxford score was 25.75 (range: 12–37) and the mean postoperative Oxford score at final follow-up was 17 (range: 11–27); the difference was significant (*P*<0.005).

There were no complications. Two patients underwent reoperation which allowed assessment of the lesion; in both cases the lesions showed good filling with fibrocartilage.

**Conclusion:**

Microfracture has been shown to be a reliable method of treatment for chondral lesions within the knee. We believe that this technique may also be applied to the shoulder; however, further study is required to assess its efficacy in this joint.

**Level of evidence:**

IV

## INTRODUCTION

Chondral lesions of the glenohumeral joint pose a significant clinical problem. The natural history of such lesions is unclear; there is no established treatment mentioned in the literature, and nonoperative treatment may not provide sufficient relief from pain. Often, Outerbridge grade IV osteochondral changes of either the glenoid or humeral articular surface may be impossible to detect with contemporary imaging techniques or clinical examination.[1–3] In these cases, the pain originating from the unrecognized osteochondral defect may be attributed to associated pathology, specifically impingement syndrome,[2] labral tears, or other concomitant intra- or extra-articular abnormalities.[4,5]

The advent of shoulder arthroscopy has enhanced the surgeon's ability to diagnose and treat abnormalities of the glenohumeral joint.[1,3,6–8] Few studies have evaluated arthroscopy in the management of grade IV chondral changes of this joint.[4] Currently, the indications, benefits, and usefulness of arthroscopy in the management of grade IV osteochondral lesions are relatively unclear.

Treatment of full-thickness chondral lesions with microfracture has become established practice for the knee joint. The intervention leads to a spontaneous repair response, which is based upon therapeutically induced bleeding from the opened subchondral bone spaces and subsequent blood clot formation.[9] This clot contains pluripotent, marrow-derived mesenchymal stem cells, which produce a fibrocartilage repair with varying amounts of type II collagen content.[10] Recent studies on the knee have shown that the functional results at 10 years are equivalent to that of autologous chondrocyte implantation.[11]

The aim of our study was to determine if microfracture was effective in the treatment of type IV chondral defects within the shoulder joint.

## PATIENTS AND METHODS

From June 2005 to November 2006, eight patients underwent shoulder arthroscopy with arthroscopic microfracture to treat full-thickness chondral lesions of less than 4 cm^2^ size. All procedures were carried out by the senior author. All patients included in the study had a postoperative diagnosis of a chondral lesion and had undergone treatment with microfracture. Only grade IV lesions (full-thickness cartilage loss with exposed subchondral bone) of less than 4 cm^2^ size were considered appropriate for treatment with microfracture. Patient data was recorded prospectively.

Chondral defects were classified according to their size and site. Microfracture of the cartilage lesions was performed according to the method described by Steadman *et al.*[12] This included debridement of the cartilage lesion to stable cartilage margins, careful removal of the calcified cartilage layer, and micropenetration of the subchondral bone using commercially available instrumentation (Linvatec, Largo, Florida). The 4-mm-wide subchondral bone bridges were carefully maintained between each microfracture hole so as to ensure the preservation of subchondral bone-plate integrity and function. Release of blood and marrow fat droplets from the microfracture holes was confirmed by eliminating arthroscopic pump pressure.

Postoperatively, the rehabilitation program was dependent on associated pathology and procedures carried out. In cases where only an isolated chondral defect was present, patients were advised to undertake immediate active movements, with avoidance of movement against resistance. Strengthening exercises began at 12 weeks.

All complications or reoperations were documented. Outcome was measured using the Constant and Oxford shoulder scores by a physiotherapist with an interest in shoulder rehabilitation. Scores were recorded preoperatively and at 3-monthly intervals. Statistical analysis was carried out using the paired Student's *t* test.

## RESULTS

The study group consisted of six men and two women. The age at surgery ranged from 27 to 55 years, with an average age of 37 years. The dominant extremity was involved in 56% of the cases. Four patients were professional rugby players.

One patient (12.5%) had an isolated chondral defect and seven patients (87.5%) had associated conditions treated simultaneously: two patients had arthroscopic subacromial decompression, two had capsular plications for multidirectional instability, and three had anterior stabilization done (one with an associated superior labrum anterior to posterior (SLAP) repair and one with repair of a small rotator cuff tear).

Five patients had humeral head defects [Figure 1a] and three had glenoid defects [Table 1 and Figure 1b].

**Figure 1a:**
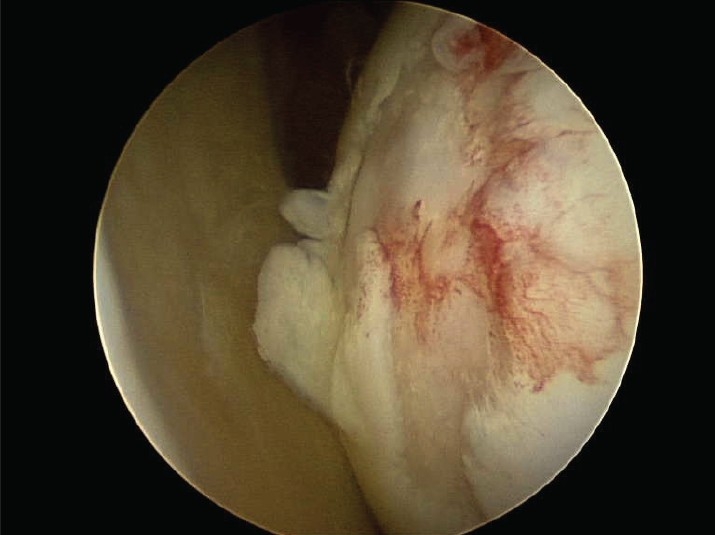
Humeral head – grade IV defect

**Table 1:** Summary of patients, associated pathology, and site of chondral lesions

Patient	Associated lesion	Age	Humeral head	Glenoid lesion
1	Bankart lesion	32		Anteroinferior and posteroinferior 1.5 × 1cm
2	SLAP tear and Bankart	27	Posteroinferior head; 2 × 2 cm	
3	None	39	Anterior head one had 1 × 1 cm	
4	Multidirectional instability – Capsular shift	33	Superomedial anterior region - 1 × 1.3 cm	
5	Subacromial impingement – Decompression	46		As follows: 2 × 2 cm osteochondral lesion in bare area (traumatic)
6	Rotator cuff tear 1 cm, Bankart	28		Osteochondral defect anterior third glenoid; 1.5 × 1 cm
7	Subacromial impingement – Decompression	55	Large central osteochondral lesion 2 × 2 cm	
8	Capsular shift for laxity	41	Superomedial anterior region - 1 × 1.3 cm	

**Figure 1b:**
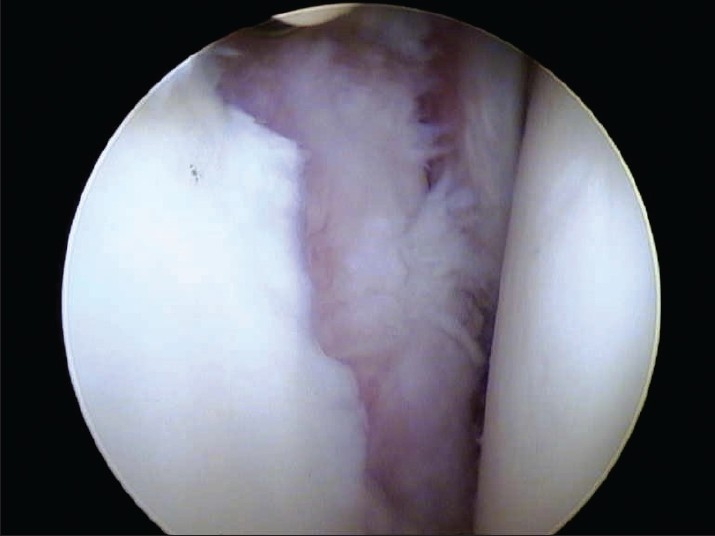
Grade IV chondral lesion of glenoid

The mean follow-up period was 15.4 months, with a range of 12–27 months. The mean preoperative Constant score was 43.88 (range: 28–70) and at final follow-up the mean Constant score was 90.25 (range: 85–100); this difference in the scores was significant (*P*<0.005). The mean preoperative Oxford score was 25.75 (range: 12–37) and the mean postoperative Oxford score at final follow-up was 17 (range: 11–27); this difference in the scores was significant (*P*<0.005). The mean preoperative range of movement was 105° (range: 60–160°) of forward flexion and 105° degrees (range: 90–150°) of abduction. Postoperatively, the mean range of movement was 176.3° (range: 150–180°) of forward flexion and 175° degrees (150–180°) of abduction. This improvement in both forward flexion and abduction was significant (*P*<0.005). Preoperatively, the mean pain score (on a scale of 0–15) was 8.5 (range; 6–15) compared to 1.25 (range: 1–2) postoperatively; this difference was significant (*P*<0.005). The mean preoperative satisfaction score (on a scale of 1–10) was 3.1 (range: 1–7) and it was 8.88 (range: 7–10) postoperatively; this difference was statistically significant (*P*<0.005).

There were no complications. Two patients underwent reoperation which allowed assessment of the lesion; one of these patients had a revision of a superior labrum anterior to posterior (SLAP) repair following another significant tackle injury, and the second patient needed a subsequent subacromial decompression. On assessment of the chondral lesions, both glenoid and humeral lesions had healed well. There was good fill and continuity of all lesions [Figures 2a and 2b].

**Figure 2a:**
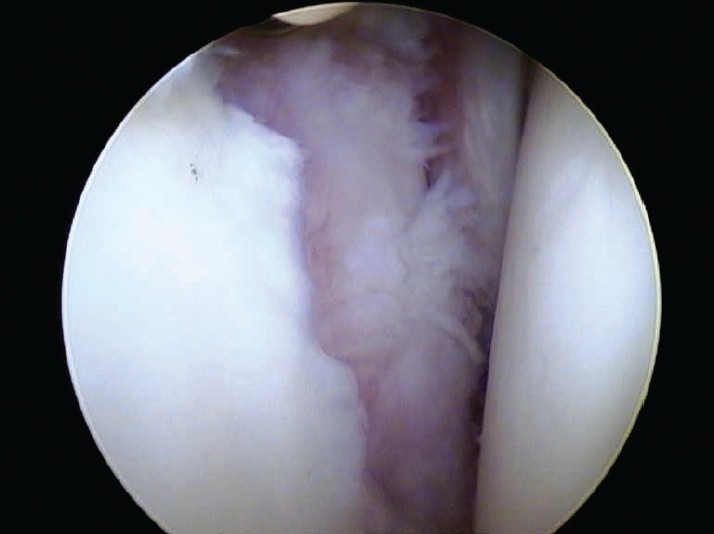
Glenoid lesion preoperatively

**Figure 2b:**
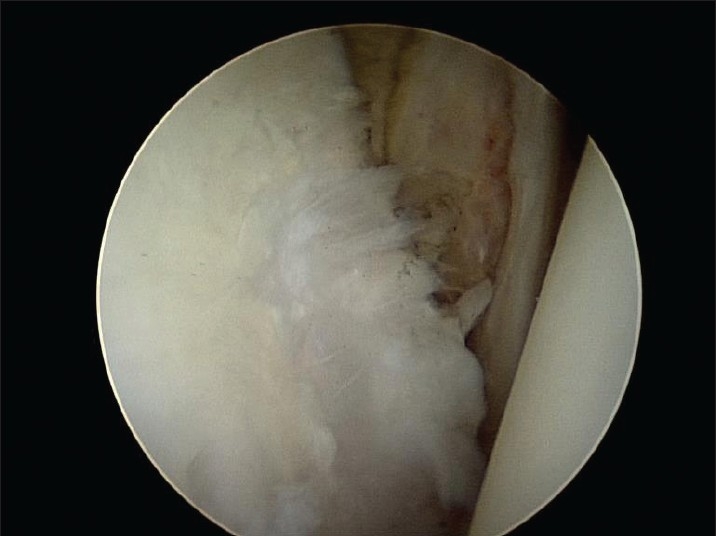
Glenoid lesion 18 months post microfracture

## DISCUSSION

It has been reported that coexisting Outerbridge grade II–IV chondral lesions are detected as incidental findings in 5% of arthroscopic shoulder procedures.[13] Various attempts have been made to treat these lesions to reduce pain and also on the assumption that such treatment will prevent continued deterioration. Preliminary studies have shown that arthroscopy may play a role in the treatment of glenohumeral osteoarthritis. Ellman[14] described grade II/III chondromalacia of the humeral head and glenoid. Arthroscopic debridement led to early pain relief in short-term follow-up. Ogilvie-Harris and Wiley[6] reported on their 10-year experience with shoulder arthroscopy; a subgroup of their patients with degenerative changes of the shoulder joint were treated with arthroscopic debridement with good results. Both Cofield[15] and Johnson[16] also reported on small groups of patients treated with arthroscopic lavage and debridement with subsequent relief of symptoms, but their results were not quantified. Weinstein[4] found that there was an improvement in pain following debridement. Postoperative function and pain levels have been shown to improve in 60–80% of patients with mild degenerative changes (Outerbridge grade I–III).[7,9] Favorable outcomes have been reported to be much lower (around 30%) when advanced degenerative changes (Outerbridge grade IV) were treated with arthroscopic debridement alone. Mean pain relief lasted 28 months. The addition of a capsular release may be of benefit in lesions less than 2 cm^2^ in size.[17]

In full-thickness cartilage lesions, repair of the lesion cannot be expected because of the limited restorative capacity of cartilage.[18–20] It has been shown within the knee that this often leads to worsening symptoms and joint deterioration.[21] Therefore, the focus of recent studies has been to find a treatment method to encourage the repair of cartilage or to produce a substitute tissue that can function as cartilage.

Urist[22] reported in 1958 that intra-articular lesions could heal by the formation of hyaline-like cartilage and it was hypothesized that the process of microfracture allows pluripotent mesenchymal cells and growth factors from the marrow space to gain access to the defect.[23,24] The scapula and humerus have an excellent vascular supply and so there appears to be no reason why this technique should not be applied to the shoulder.

The technique of microfracture is ideally suited to the shoulder. It is minimally invasive because it is arthroscopic through standard portals. In contrast to abrasion chondroplasty, the subchondral bone plate is not completely destructed but is partially preserved between the microfracture holes, improving load-bearing ability following healing.[25] In contrast to Pridie drilling, microfracture avoids any heat necrosis or polishing of the subchondral bone.[26,27] The equipment is standardized and the costs are minimal since expensive cell cultures are not necessary. Unlike osteochondral, perichondral, periosteal, or chondral autograft procedures, the problems of harvest site morbidity and arthrotomy are avoided.[28]

The hemarthrosis resulting from the microfracture procedure may have further benefits. It is generally accepted that meniscal repair healing is better when carried out in association with anterior cruciate ligament reconstruction.[29] It is thought that the pluripotent cells may improve the repair process. Given that chondral lesions in the shoulder commonly occur in association with other pathology, there may be potential for improved healing of simultaneous labral or rotator cuff repairs.

It is difficult to quantify the effect of microfracture on patient outcomes in this study. All but one patient had associated pathology with simultaneous procedures carried out. Thus one would, in any case, expect improvement in pain and function following surgery. The lone patient with an isolated chondral defect improved significantly postoperatively, especially with respect to pain. Reoperation on two patients demonstrated evidence of successful fibrocartilage regeneration. It would appear reasonable to assume that this regrowth contributed, at least in part, to pain reduction and functional improvement in our patients. Regenerating fibrocartilage, it is hoped, will provide more prolonged symptomatic relief as compared to debridement.

Similar results have been shown by Siebold *et al.*[30] in 2003. They treated five humeral head lesions with a combination of microfracture and periosteal flap using an open technique. The Constant score increased from 43% preoperatively to 82% postoperatively. At second-look arthroscopy the size of all the lesions had decreased. The addition of the periosteal patch may have added little to their success.

There are a number of weaknesses in our study. One criticism could be with regard to the outcome tools that we used. The Constant and Oxford shoulder scores are global outcome measures and are not specific for chondral lesions. However, the Constant and Oxford scores are established scores and have been shown to have good correlation with shoulder impairment.

The mean follow-up period is relatively short at 15.4 months. Thus, while our follow-up is sufficient to show initial improvement, it remains to be seen if this improvement will be maintained.

We did not obtain postoperative follow-up MRI scans in our patients. Preoperatively, only 2 patients had a MRI. Both these patients were professional rugby players and had moved to another location, thus they were not available for follow-up.

A further weakness of our study could be that we did not obtain biopsies. We, however, felt that this would add little to our study as the histology of the regenerate following microfracture is well documented.

We only had eight patients in our case series. These lesions are relatively uncommon and thus it is difficult to obtain large numbers without multicenter collaboration.

We believe that microfracture is a useful technique in the shoulder, and may prevent the natural deterioration of grade IV lesions which is seen in other joints. Isolated grade IV chondral lesions are a rare finding at arthroscopy and therefore it would be difficult to study these lesions exclusively. Multicenter, prospective randomized controlled trials are needed to fully investigate the effect of microfracture within the shoulder. Long-term follow-up is necessary to determine if continued pain relief, beyond that obtained with debridement alone, can be achieved via means of fibrocartilage regeneration.

## References

[1] Cofield RH (1983). Arthroscopy of the shoulder. Mayo Clin Proc.

[2] Cofield RH, Frankle MA, Zuckerman JD (1995). Humeral head replacement for glenohumeral arthritis. Semin Arthroplasty.

[3] Ellman H, Harris E, Kay S (1992). Early degenerative joint disease simulating impingement syndrome: arthroscopic findings. Arthroscopy.

[4] Weinstein D, Bucchieri J, Pollock R, Flatow E, Bigliani LU (2000). Arthroscopic debridement of the shoulder for osteoarthritis. Arthroscopy.

[5] Ogilvie-Harris DJ, Wiley AM (1986). Arthroscopic surgery of the shoulder. A general appraisal. J Bone Joint Surg Br.

[6] Ogilvie-Harris DJ, Wiley AM (1986). Arthroscopic surgery of the shoulder. A general appraisal. J Bone Joint Surg Br.

[7] Ogilvie-Harris DJ (1987). Arthroscopy and arthroscopic surgery of the shoulder. Semin Orthop.

[8] Ogilvie-Harris DJ, D'Angelo G (1990). Arthroscopic surgery of the shoulder. Sports Med.

[9] Brittberg M, Lindahl A, Nilsson A, Ohlsson C, Isaksson O, Peterson L (1994). Treatment of deep cartilage defects in the knee with autologous chondrocyte transplantation. N Engl J Med.

[10] Thomas L, Wickiewicz, Robert G Marx, Kai Mithoefer, Riley J Williams, Russell F Warren, Hollis G Potter, Christopher R Spock, Edward C Jones (2005). Lesions in the Knee. A Prospective Cohort StudyThe Microfracture Technique for the Treatment of Articular Cartilage JBJS Am.

[11] Steadman JR, Briggs KK, Rodrigo JJ, Kocher MS, Gill TJ, Rodkey WG (2003). Outcomes of microfracture for traumatic chondral defects of the knee: average 11-year follow-up. Arthroscopy.

[12] Steadman JR, Rodkey WG, Rodrigo JJ (2001). Microfracture: surgical technique and rehabilitation to treat chondral defects. Clin Orthop Relat Res.

[13] Iannotti JP, Naranja RJ, Warner JJP, Warner JJP, Iannotti JP, Gerber C (1997). Surgical management of shoulder arthritis in the young and active patient. Complex and revision problems in shoulder surgery.

[14] Ellman HA (1987). Arthroscopic subacromial decompression: Analysis of 1-3 year results. *Arthroscopy*.

[15] Cofield RH (1983). Arthroscopy of the shoulder. *Mayo Clin Proc*.

[16] Johnson CC (1987). The shoulder joint: An arthroscopic perspective of anatomy and pathology. *Clin Orthop*.

[17] Cameron BD, Galatz LM, Ramsey ML, Williams GR, Iannotti JP (2002). Non-prosthetic management of grade IV osteochondral lesions of the glenohumeral joint. J Shoulder Elbow Surg.

[18] Mankin HJ (1982). The response of articular cartilage to mechanical injury. J Bone Joint Surg Am.

[19] Breinan HA, Martin SD, Hsu HP, Spector M (2000). Healing of canine articular cartilage defects treated with microfracture, a type-II collagen matrix, or cultured autologous chondrocytes. J Orthop Res.

[20] Breinan HA, Minas T, Hsu HP, Nehrer S, Sledge CB, Spector M (1997). Effect of cultured autologous chondrocytes on repair of chondral defects in a canine model. J Bone Joint Surg Am.

[21] Messner K, Maletius W (1996). The long-term prognosis for severe damage to weight-bearing cartilage in the knee: A 14-year clinical and radiographic follow-up in 28 young athletes. Acta Orthop Scand.

[22] Urist MR (1958). The repair of articular surfaces following arthroplasty of the hip. Clin Orthop.

[23] Steadman JR, Rodkey WG, Briggs KK, Rodrigo JJ (1999). [The microfracture technique in the management of complete cartilage defects in the knee joint]. Orthopade.

[24] Frisbie DD, Oxford JT, Southwood L (2003). Early events in cartilage repair after subchondral bone microfracture. Clin Orthop.

[25] Menche D, Frenkel S, Blair B, Watnik N, Toolan B, Yahhoubian R (1996). A comparison of abrasion arthroplasty and subchondral drilling in the treatment of full-thickness cartilage lesions in the rabbit. Arthroscopy.

[26] Pridie KH (1959). A method of resurfacing osteoarthritic knee joints. J Bone Joint Surg Br.

[27] Tippet JW, McGinty J (1991). Articular cartilage drilling and osteotomy in osteoarthritis of the knee. *Operative Arthroscopy*.

[28] Sledge SL (2001). Microfracture techniques in the treatment of osteochondral injuries. Clin Sports Med.

[29] Cannon WD, Vittori JM (1992). The incidence of healing in arthroscopic meniscal repairs in anterior cruciate ligament-reconstructed knees versus stable knees. Am J Sports Med.

[30] Siebold R, Lichtenberg S, Habermeyer P (2003). Combination of microfracture and periostal-flap for the treatment of focal full thickness articular cartilage lesions of the shoulder: A prospective study. Knee Surg Sports Traumatol Arthrosc.

